# UK Reproducibility Network open and transparent research practices survey dataset

**DOI:** 10.1038/s41597-024-03786-z

**Published:** 2024-08-23

**Authors:** Lukas Hughes-Noehrer, Noémie Aubert Bonn, Marcello De Maria, Thomas Rhys Evans, Emily K. Farran, Laura Fortunato, Emma L. Henderson, Neil Jacobs, Marcus R. Munafò, Suzanne L. K. Stewart, Andrew J. Stewart

**Affiliations:** 1 University of Manchester, Department of Computer Science, Manchester, M13 9PL United Kingdom; 2 University of Reading, Department of Agri-Food Economics and Marketing, Reading, RG6 6AH United Kingdom; 3 University of Greenwich, School of Human Sciences, London, SE10 9LS United Kingdom; 4 University of Surrey, School of Psychology, Guildford, GU2 7XH United Kingdom; 5 University of Oxford, Institute of Human Sciences, Oxford, OX2 6PE United Kingdom; 6 Santa Fe Institute, Santa Fe, New Mexico 87501 United States of America; 7 University of Bristol, School of Psychological Science, Bristol, BS8 1TU United Kingdom; 8 University of Chester, School of Psychology, Chester, CH1 4BJ United Kingdom

**Keywords:** Research management, Policy, Institutions, Education, Research data

## Abstract

Openness and transparency in the research process are a prerequisite to the production of high quality research outputs. Efforts to maximise these features have substantially accelerated in recent years, placing open and transparent research practices at the forefront of funding and related priorities, and encouraging investment in resources and infrastructure to enable such practices. Despite these efforts, there has been no systematic documentation of current practices, infrastructure, or training and resources that support open and transparent research in the UK. To address this gap, we developed and conducted the Open and Transparent Research Practices survey, a large-scale audit study completed by research-active staff in UK research institutions to better understand existing practices, needs, support, and barriers faced when implementing open and transparent research. The data presented here capture responses from over 2,500 research-active staff based at 15 institutions affiliated with the UK Reproducibility Network. The data provide a snapshot of open research practices that can be used to identify barriers, training needs, and areas that require greater investments.

## Background & Summary

In the past decade, efforts to cultivate open and transparent research have shifted from incremental and local initiatives to national and global activity. For example, in 2018, cOAlition S brought together national research agencies, funders, and other organisations from Europe and the rest of the world in adopting Plan S, which requires that all scientific publications resulting from publicly funded research be made immediately openly accessible upon publication^1^. In the same period, national and international funders across Europe also voiced their expectations that researchers share their data openly whenever possible, leading to the development of infrastructures such as the European Open Science Cloud (EOSC), OpenAIRE, and the publishing venue *Open Research Europe*. At a global level, the *UNESCO Recommendation on Open Science*^2^, released in 2021, formalised common standards on a global scale, prompting countries around the world to initiate reforms in this direction.

The UK has been a driving force in these broad, international efforts. This is not surprising considering that formal discussions on open research within the UK began nearly 20 years ago. Early discussions focused largely on open access, leading, in 2005, to the release of the first Open Access Policy from the Research Councils and from independent research funders such as the Wellcome Trust^3^. Since then, periodic evidence-based recommendations and revisions of the Open Access Policies have been carried out, leading to important evidence on how UK-funded research findings can be made more accessible^4–6^. The RCUK Open Access policy was carried on and expanded by UK Research and Innovation (UKRI, an umbrella organisation which coordinates seven government-funded research councils, Research England, and Innovate UK to support research and innovation in the UK) with a requirement that all outputs of the research it funds can be openly accessed, viewed, and downloaded without restriction and at no cost. Adding to this focus on open access, the UK has also led important initiatives on the broader elements of open research. In 2023, the House of Commons Science, Innovation, and Technology Committee published a report on its inquiry into reproducibility and research integrity launched in July 2021, recommending greater adoption of open and transparent practices^7^. UK funders have also adopted strong data sharing policies. This includes the 2011 EPSRC policy framework on research data that was adopted by several other funders throughout the UK, and the Concordat on Open Research Data^8^ launched in 2016. The latter provides ten principles which outline responsibilities for good data practice in order to maximise the availability of research data, software, and materials with as few restrictions as possible.

In addition to these national and organisational initiatives, UK-based researchers have led research into the importance of open and transparent research for the advancement of knowledge and for its impact on society. Discussions on research waste^9^ and on the reproducibility crisis^10^, for example, led to establishment of the UK Reproducibility Network (UKRN; www.ukrn.org) in 2019, a national peer-led consortium that seeks to understand the factors that contribute to poor research quality and to develop approaches to counter these^11,12^.

Such initiatives place openness and transparency at the forefront of national research priorities, increasing awareness and encouraging investment in resources and infrastructures to enable open research. To inform these investments, it is important to understand the practices and infrastructures already in place, and what is still needed, to support open and reproducible research. A few large-scale projects have provided evidence in this area. For example, in 2019, the European Commission released the final report from the Open Science Monitor^13^, a project which captured practices, attitudes, and barriers to open research across Europe. The findings from the project provide data from a broad diversity of indicators of open research across several countries, enabling large scale comparisons and providing an overview of progress in a wide range of open research practices, including open data, open collaborations, and open access. The Open Science Observatory^14^ - an initiative from OpenAIRE - also provides useful and current information on open access, open datasets, open software, and other research outputs. However, the Open Science Observatory is captured through bibliometrics, limiting the overall depth of interpretations that can be made about barriers, needs, and drivers for open research practices. In 2022, members of the UKRN published the results of the Brief Open Research Survey (BORS)^15^, which measured awareness and uptake of Open Research practices across the UKRN Local Networks. The survey found that respondents were most aware of Open Access publications, preprints and open data, and the most commonly reported means to foster further uptake of Open Research practices were incentives, dedicated funding, and recognition in promotion and recruitment criteria. More recently, a survey conducted across psychology departments in the UK and Ireland shed light on the engagement of their researchers with open research practices^16^. The survey provides useful insights into the topic but is limited by its disciplinary boundary. To address these apparent gaps and provide information about the UK landscape of open research from the perspective of research-active staff across all disciplines, we developed and conducted the Open and Transparent Research Practices (OTRP) survey, a large-scale study of research-active staff in UK research institutions to better understand self-reported practices, needs, support, and barriers faced in implementing open and transparent research practices. The data presented here capture responses from research-active staff based at 15 institutions with a UKRN Institutional Lead (IL).

The survey questions are divided into 14 topics covering a range of open research practices. The 14 practice areas were selected based on consensus among an IL committee recruited from partaking institutions and defined according to the terminology used in the Framework for Open and Reproducible Research Training (FORRT) community-sourced glossary^17^. The practices are (i) research co-production; (ii) conduct of open research consistent with relevant legal, ethical and regulatory constraints; (iii) transparent qualitative data; (iv) data management; (v) pre-registration of research protocols; (vi) use of open source software; (vii) creation of open source software; (viii) version control of research products; (ix) computational reproducibility of data analysis; (x) sharing of data, code, or other evidence according to the FAIR principles; (xi) guidelines for recognising the specific substantive contribution of everyone involved in research projects; (xii) declaration of interests; (xiii) publication of preprints; and (xiv) open access publishing. For each topic, the survey assessed respondents’ own practices; perceived importance of the topic; available support, help, and training; and potential barriers to undertaking the practices in their institution.

The data presented in this descriptor can be used to identify needs and barriers in embedding open and transparent research practices in UK institutions. The data produced have high reuse potential for identifying areas that require greater investments, for highlighting barriers to open and transparent research practices, and for identifying training needs in the topics investigated.

## Methods

The OTRP survey was conducted online using the Qualtrics platform^18^ between December 2022 and April 2023 and was open to all UKRN member institutions with an IL that wanted to participate (15 institutions chose to take part). Institutions were able to choose suitable time windows within this period to avoid clashes with other institution-wide surveys as much as possible. Participants were recruited locally via UKRN Institutional Leads or their representatives, and were either recruited via opportunity sampling or via a stratified sampling strategy. Opportunity sampling included sending out cascading invitations containing a survey link to all faculties and sub-units (e.g., schools and departments), whilst stratified sampling involved sending targeted invitations to named individuals meeting specific criteria. Stratified sampling was chosen by two institutions as it was a priority for their organisation to obtain a representative sample to assess prevalence and reduce self-selection bias as much as possible. For a list of all partaking institutions, participant numbers, sampling strategies, and the dates of first and last response per institution, please refer to Table 1.

**Table 1:** Partaking institutions (in alphabetical order), participant numbers, sampling approach, and first and last date of response [dd/mm/yy].

Institution	Number of respondents	Sampling	First and last response date
King’s College London	148	Opportunity	17/01/23–12/04/23
Newcastle University	125	Opportunity	22/12/22–02/03/23
Northumbria University	98	Opportunity	01/02/23–28/04/23
Oxford Brookes	163	Opportunity	12/12/22–30/01/23
University College London	693	Opportunity	20/12/22–02/03/23
University of Bristol	57	Stratified	11/01/23–07/03/23
University of Glasgow	80	Opportunity	25/01/23–10/04/23
University of Liverpool	67	Opportunity	01/03/23–17/04/23
University of Manchester	303	Opportunity	13/12/22–25/03/23
University of Oxford	129	Opportunity	12/12/22–10/04/23
University of Reading	81	Stratified	23/03/23–13/04/23
University of Sheffield	206	Stratified	18/01/23–17/04/23
University of Southampton	143	Opportunity	15/02/23–09/04/23
University of Surrey	214	Opportunity	12/12/22–17/04/23
**Total**	**2,567**		

Participants were asked to respond to every question in the survey. Out of 2,567 submissions, 511 (20%) participants returned fully completed surveys and a further 670 (26%) participants answered at least 50%.

This study was incentivised. Every participant had the chance to enter a prize draw at the end of the study via a separate survey form to win one of ten £50.00 (Fifty Pound) Amazon vouchers (10 vouchers per partaking institution). The identifiable information collected to take part in the prize draw was not linked to the OTRP Survey to safeguard anonymity.

### Survey development

The survey instrument was developed through a common effort of UKRN member institutions and the final questionnaire was reviewed and approved by UKRN Institutional Leads. The survey was user-tested by ten individuals at selected institutions (i.e., University of Hull, University of Oxford, Northumbria University, Brunel, University of Greenwich, University of Leeds, University of Chester, University of Manchester, and University of Bristol), which were approached on account of their experience with the design of surveys and sampled from a variety of backgrounds. The final survey was revised according to their comments. The survey is split into three sections.

#### Section 1

Section 1 contains demographic questions split in two parts. Part 1, “Institution and Career”, asks participants about how long they have been at their current institution, their research discipline, their current job role and career stage, and the research methods they mostly use to conduct their research (i.e., qualitative, quantitative, mixed methods, or other). Part 2, “Protected Characteristics”, asks participants about their gender and ethnicity to monitor equality, diversity, and inclusion.

The question about research discipline featured a drop-down menu listing entries in the top-level tier of the Common Aggregation Hierarchy (CAH), a standardised taxonomy of subject codes and terms^19^. In the version of the survey circulated at the University of Oxford, this question was replaced with one on primary affiliation (equivalent to Q1 in^20^, with “Faculty of Asian and Middle Eastern Studies” in place of “Faculty of Oriental Studies”, and “Department of Biology” in place of “Department of Zoology” and “Department of Plant Sciences”). In the final dataset, we translated Oxford primary affiliations to CAH to ensure that they could be analysed with the full dataset. Correspondences were agreed upon between N.A.B. and L.F. A key of correspondence between these primary affiliations and CAH categories is available on Figshare (see Data Records below).

The question about career stage used the following categorisation: *Phase 1: Junior (e.g., PhD candidate, Research Assistant)*, *Phase 2: Early (e.g., Research Associate, first grant holder, Lecturer)*, *Phase 3: Mid/Recognised (e.g., Senior Lecturer, Reader, Senior Researcher)*, *Phase 4: Established/Experienced (e.g., Professor, Principal Fellows or Scientists)*^21^. In one institution, this question was replaced with one on staff roles as defined by the University’s Human Resources Department. Given the complexity of equivalence between these two categories, we left this column blank for respondents from this institution and explain this change in the codebook.

#### Section 2

Section 2 contains the main corpus of the questionnaire and surveys the 14 selected open research practices. A comprehensive list of all practices and a link to the relevant FORRT Glossary entries is available in Table 2.

**Table 2:** Description of the 14 practices included in the survey.

Practice	Description	Link
Research co-production	“An approach to research where stakeholders who are not traditionally involved in the research process are empowered to collaborate, either at the start of the project or throughout the research lifecycle.”	^30^
Conduct of open research consistent with relevant legal, ethical and regulatory constraints	Conducting research following the legislation and standards that dictate ethical and lawful research practices. These legislation and standards may vary between countries and settings.	^31^
Transparent qualitative data practices	Ensuring that qualitative research - “Research which uses non-numerical data, such as textual responses, images, videos or other artefacts, to explore in-depth concepts, theories, or experiences” - is conducted in an open and accessible manner, enabling external evaluation.	^32^
Defining the data, code, or other evidence before the start of data collection and analysis	Defining the data, code, or other evidence on which research findings will be based on and how this will be managed and shared before the start of data collection and analysis (e.g., Data Management Plans).	^33^
Pre-registration of research protocols	“The practice of publishing the plan for a study, including research questions/hypotheses, research design, data analysis before the data have been collected or examined.”	^34^
Use of open source software created by others	Using “computer software in which source code is released under a license that permits others to use, change, and distribute the software to anyone and for any purpose.”	^35^
Creation of open source software	Creating “computer software in which source code is released under a license that permits others to use, change, and distribute the software to anyone and for any purpose.”	^35^
Version control of research products	“The practice of managing and recording changes to digital resources (e.g. files, websites, programmes, etc.) over time so that you can recall specific versions later.”	^36^
Computational reproducibility of data analysis	“Ability to recreate the same results as the original study (including tables, figures, and quantitative findings), using the same input data, computational methods, and conditions of analysis.”	^37^
Preparing data, code, or other evidence according to the FAIR principles	Preparing data, code, or other evidence in a way to ensure that it is Findable, Accessible, Interoperable and Reusable (FAIR).	^38^
Guidelines for recognising the specific substantive contribution of everyone involved in research projects	Use of guidelines such as the CRediT taxonomy, community guidelines, models, FAIRsharing, etc. to recognise specific substantive contribution of everyone involved in research projects. *Note: This practice included a mouse hover with examples of guidelines* *“CRediT taxonomy, community guidelines, models, FAIRsharing, etc.”*	N/A
Declaring conflicts of interests	Declaring any “financial or non-financial relationship, activity or other interest that might compromise objectivity or professional judgement on the part of an author, reviewer, editor, or editorial staff.”	^39^
Publication of pre-prints	“A publicly available version of any type of scientific manuscript/research output preceding formal publication, considered a form of Green Open Access. Preprints are usually hosted on a repository (e.g. arXiv) that facilitates dissemination by sharing research results more quickly than through traditional publication.”	^40^
Ensuring publications are Open Access (OA)	“Free availability of scholarship on the public internet, permitting any users to read, download, copy, distribute, print, search, or link to the full texts of these research articles, crawl them for indexing, pass them as data to software, or use them for any other lawful purpose, without financial, legal, or technical barriers other than those inseparable from gaining access to the internet itself.”	^41^

When introduced in the survey, the practices were accompanied by a link to the FORRT glossary to provide further information about the topic of interest.

**Fig. 1:**
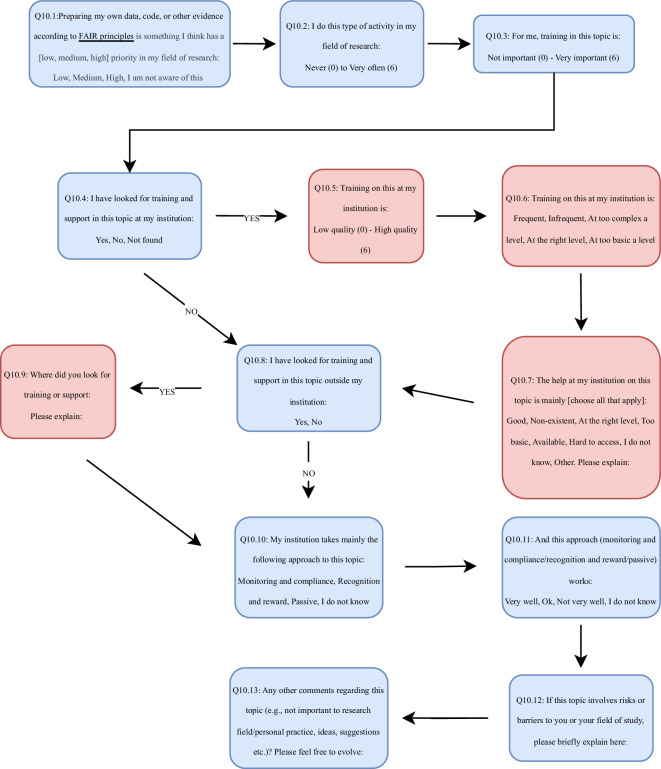
Example question structure using Question 10 about FAIR sharing practices.

All practices were surveyed using the same structure and wording of questions. To showcase this, an example question structure with the answer logic can be found in Fig. 1. Respondents were asked about the following elements:

1. Whether this practice has a low, medium, or high priority in their field of research;
2. How often they do this type of activity in their field of research;
3. How important training in this topic is for them;
4. Whether they have looked at training and support in this topic at their institution, and if yes, they were asked for additional indicators of quality, frequency, and complexity level of training on this topic at their institution as well as the value and accessibility of the help on this topic at their institution;
5. Whether they have looked for training (e.g., courses, workshops) in this topic outside their institution, and if yes, where they have looked for training and support;
6. Which approach their institution mainly takes towards this topic (i.e., monitoring and compliance, recognition and reward, passive, or “I do not know”);
7. How well the approach taken in their institution works;
8. Whether this topic involves risks or barriers to them or their field of study;
9. Whether they had any comments regarding this topic (e.g., not important to research field/personal practice, ideas, suggestions etc.).

#### Section 3

Section 3 contains more general questions regarding open research and the perspectives of respondents on open research. It asks respondents how likely they are to recommend the adoption of open and transparent research practices to colleagues (0-6 rating) and to select what they consider to be the greatest barrier to the uptake of open research practices from 10 options.

#### Optional supplementary questions

Following this content, such that it would not affect responses to the preceding questions, institutions were supported to include any other additional questions that would be helpful to guide local action. Surrey, Oxford, and Keele added such questions and these are available to view in the project materials document (see ‘Data Records’ below for specific links). The data collected from these questions are not shared within the current project as it may lead to identification of the institutions of participants and are not considered to have high re-use potential beyond the specific institution.

### Ethical approval and other logistics

The project received ethics approval from the University of Manchester Proportionate Research Ethics Committee (Ref: 2022-15389-26232). The Application for Ethics Approval as well as the Letter of Approval from the University of Manchester are available on Figshare^22^. To be able to waive the necessity of ethical approval by every partaking institution and to share data, every institution had to sign a Letter of Collaboration to ensure that the relevant data protection training has been completed and no further ethical review will be required. Thirteen signed letters of collaboration were received, with the exception of Surrey which decided to apply for internal approval as well after having added a considerate amount of Surrey-specific questions to the main survey.

Inclusion criteria were communicated in recruitment materials and reiterated on the survey’s welcome page before we asked for consent. ILs were given instructions to distribute the survey among “research active staff across institutions”, and informed that “this ranges from PGRs to professorial grades and should include ALL subjects/departments at [their] university.” Further, information given prior to participation stated that everyone is encouraged to take part whether or not they are aware of open research practices, and whether or not they deem them relevant or necessary for their fields of research.

Participants provided consent to take part in the survey by agreeing to a consent statement included at the beginning of the survey. The consent statement linked to the Participant Information Sheet, which provided further details on participation and on the scope of the project. The Participation Information Sheet and consent question are available in the materials provided (see ‘Data Records’ below for specific links). Participation was voluntary, and participants could close the browser at any time to exit the survey. However, they were informed that collected data could not be removed after it was submitted in the study as participation was anonymous and, therefore, data could not be attributed to individuals. Participants who entered the prize draw were able to enter their personal details on a separate survey which was not linked to any of the OTRP survey data.

## Data Records

The dataset is available in CSV format on Figshare^22^ (file ukrn_otrp_survey_data.csv) under a CC-BY 4.0 licence. To minimise risks of re-identification of participants, the dataset does not include details about institutional affiliation. Aggregated data on institutional affiliation can be found in Table 1. A codebook which describes each of the dataset question details, descriptions, and levels of processing (file Codebook.csv), and the equivalence key that was used to translate the primary affiliation of The University of Oxford to CAH are also available on Figshare (file Oxford-CAH_EquivalenceKey.xlsx). A complete printout of the questionnaire in PDF format is available on the same Figshare repository (file ukrn_otrp_survey_questionnaire.pdf).

To provide context and transparency on the research process that led to the data, the Figshare folder also includes the Data Management Plan (file ukrn_otrp_survey_dmp_v1.0.pdf), the documents used in the ethics submission to the University of Manchester Research Ethics Committee in PDF format (Letter of Approval, file letter_ethics_approval.pdf and Ethics Application document file letter_ethics_approval.pdf), the Participation Information Sheet in PDF format (file ukrn_otrp_survey_pis_v1.1.pdf) that was available to participants before giving consent, and the sample communication templates that were used by UKRN Institutional Leads to invite participants in PDF format (file ukrn_survey_IL_comms.pdf).

## Technical Validation

The dataset was checked for validity by several authors to ensure reliability of the collected data and anonymity of respondents. Data range and data type checks were performed as the data validation process. Data validation checks include ensuring that every column contains the data type we expect (e.g., numerical or string), checks for total number of observations (number of rows consistent with responses), data range checks (i.e., checking values in scale-type questions if they reflect the possible range of numbers), and checking for unexpected missing values. We also applied built-in validation functionalities provided by Qualtrics to restrict answers to single choice options where just one answer was appropriate. Tests were performed on each institutional dataset and on the aggregated dataset this descriptor is referring to. Furthermore, the analysis script was tested for reproducibility and data integrity. No information gathered through the survey questionnaire was altered or removed, except institutional affiliations and institution-specific answers as indicated in the equivalence key and the codebook on Figshare.

### Considerations for reuse

The data captures perspectives from research-active staff with regards to open research practices and institutional support and approaches for such practices. As such, it provides an indication of current trends and needs, and evidences self-reported practices. Self-reported practices may differ from actual practices for a number of reasons, and individuals reusing the data should be aware of this possibility and reflect it in their work. In fact, previous research on responsible research practices suggests that researchers are likely to under-report their own negative research practices (e.g., questionable research practices)^23^, and research on the incidence of responsible research practices has started using ’truth-telling’ incentives to address the problem^24^. Since our survey addressed open research practices, which can be associated with responsible research practices, it is possible that the opposite phenomena occurred, namely that respondents over-reported their engagement with open research practices.

Individuals reusing the data should also be mindful of the sampling strategy used in this survey and ensure that interpretations reflect the methodology used to capture the data. While the data can provide relevant information about specific open and transparent research practices, they should not be used to generalise answers to the perspective of all UK researchers nor of the institutions involved. Given the sampling strategy, we cannot exclude the possibility that responses were subject to participation bias (e.g., individuals with a prior interest or a prior frustration with open research practices may have been more prone to participate, such that responses may be systematically different from the views of individuals who chose not to respond). We also want to acknowledge possible bias inherent in the way the survey was designed and whilst we aimed to be comprehensive, given the wide variety of practices and how these practices are implemented and understood in different disciplines, it is likely impossible to capture all variations. In addition, the number of respondents varied between responding institutions and given the importance of institution-specific questions (e.g., regarding training and support available), data reuse should be careful of generalisations. The data should also be considered within their context of UK research institutions and may not be generalisable to other countries.

The high prevalence of incomplete responses should also be considered and reflected transparently in future analyses and interpretation of the data. Appropriate checks should be undertaken to ensure that the data can be used to compare or correlate answers with demographic characteristics.

Despite these considerations, respondents were fairly balanced in terms of gender (44.4% Men, 50.2% Women, 1.1% Non-binary, 3.5% Prefer to not disclose, and 0.8% Prefer to self-describe) and career stage, although slightly skewed towards early career (28.3% Phase 1: Junior, 32.0% Phase 2: Early, 20.1% Phase 3: Mid/Recognised, and 19.6% Phase 4: Established/Experienced). Responses were also spread across a broad spectrum of disciplines, with a slightly larger proportion of respondents from CAH02 (Subjects allied to medicine), CAH03 (Biological and sport sciences), CAH10 (Engineering and technology), CAH15 (Social sciences), CAH04 (Psychology), and CAH01 (Medicine and dentistry). Most respondents used quantitative (42.6%) or mixed methods (35.4%) in their research compared to qualitative methods (19.4%) or other methods (2.7%). Three-quarters of respondents identified as white (75.9%), 12.4% as Asian or Asian-British, 2.2% as Black, Black-British, Caribbean or African, 3.7% as Mixed or multiple ethnic groups, and 5.9% as Other ethnic group.

## Data Availability

The dataset can be used for quantitative and qualitative analysis methods. Researchers who wish to run an already-established quantitative analysis script can find the relevant Python code on Figshare^25^. The analysis code is openly available and free to use under GPL 3.0+. As listed in the requirements file, the script uses *NumPy*^26^ and *pandas*^27^ for statistical analysis and *matplotlib*^28^ with *seaborn*^29^ to visualise the data. However, using these packages is not mandatory as the CSV format allows for statistical analysis with any statistical software.
