# Author Correction: UK Reproducibility Network open and transparent research practices survey dataset

**DOI:** 10.1038/s41597-024-03955-0

**Published:** 2024-10-17

**Authors:** Lukas Hughes-Noehrer, Noémie Aubert Bonn, Marcello De Maria, Thomas Rhys Evans, Emily K. Farran, Laura Fortunato, Emma L. Henderson, Neil Jacobs, Marcus R. Munafò, Suzanne L. K. Stewart, Andrew J. Stewart

**Affiliations:** 1 University of Manchester, Department of Computer Science, Manchester, M13 9PL United Kingdom; 2 University of Reading, Department of Agri-Food Economics and Marketing, Reading, RG6 6AH United Kingdom; 3 University of Greenwich, School of Human Sciences, London, SE10 9LS United Kingdom; 4 University of Surrey, School of Psychology, Guildford, GU2 7XH United Kingdom; 5 University of Oxford, Institute of Human Sciences, Oxford, OX2 6PE United Kingdom; 6 Santa Fe Institute, Santa Fe, New Mexico 87501 United States of America; 7 University of Bristol, School of Psychological Science, Bristol, BS8 1TU United Kingdom; 8 University of Chester, School of Psychology, Chester, CH1 4BJ United Kingdom

**Keywords:** Research management, Policy, Institutions, Education, Research data

Correction to: *Scientific Data* 10.1038/s41597-024-03786-z, published online 23 August 2024

In the version of the article initially published, in the “Sampling” column of Table 1, the University of Sheffield was originally listed as “Opportunity” but has now been amended to “Stratified”. Additionally, in the fourth paragraph of the “Background & Summary” section, the text “In 2022, members of the UKRN published the results of the Brief Open Research Survey (BORS), which measured awareness and uptake of Open Research practices across the UKRN Local Networks. The survey found that respondents were most aware of Open Access publications, preprints and open data, and the most commonly reported means to foster further uptake of Open Research practices were incentives, dedicated funding, and recognition in promotion and recruitment criteria” has been added, alongside a new ref. 15: Norris, E. *et al*. Development of the brief open research survey (bors) to measure awareness and uptake of open research practices, 10.31222/osf.io/w48yh (2022). These corrections have been made to the HTML and PDF versions of the article.

